# Osteoporosis and Sarcopenia Increase Frailty Syndrome in the Elderly

**DOI:** 10.3389/fendo.2019.00255

**Published:** 2019-04-24

**Authors:** Emanuela A. Greco, Peter Pietschmann, Silvia Migliaccio

**Affiliations:** ^1^Section of Medical Pathophysiology, Endocrinology and Food Science, Department of Experimental Medicine, University of Rome Sapienza, Rome, Italy; ^2^Department of Pathophysiology and Allergy Research, Center of Pathophysiology, Infectiology, and Immunology, Medical University of Vienna, Vienna, Austria; ^3^Unit of Endocrinology, Section of Health Sciences, Department of Movement, Human and Health Sciencies, University of Rome Foro Italico, Rome, Italy

**Keywords:** osteoporosis, sarcopenia, obesity, frailty syndrome, aging, gender, physical activity, diet

## Abstract

Musculoskeletal aging is a major public health interesting and strain due to the significant demographic modifications in the population, and it is linked to high risk of falls, loss of autonomy in elderly individuals and institutionalization with small health outcomes. Thus, this pathological status is related to high morbidity and health care rates. Bone mass and muscle mass and strength increase during late adolescence and early adulthood but start to reduce noticeably from the fifth decade of life and are closely linked. Bone and muscle tissues were increasingly recognized, as endocrine target organs and endocrine organs themselves, interacting through paracrine and endocrine signals. During growth, bone mineral content closely correlates with muscle mass, and several evidences suggest that osteoporosis and sarcopenia present common pathophysiological factors and show the correlation between low bone mineral density and sarcopenia in both men and women. Then, sarcopenia and osteoporosis, typical features of aging, are often associated with each other and with the frailty syndrome. In particular, sarcopenia and osteoporosis are major contributors to disability and frailty and the common denominators are age-related chronic inflammation, changes in body composition and hormonal imbalance. Frailty syndrome is characterized by a reduced response to stress, triggering the decline of the physiological functioning of the various systems. Frailty syndrome, typical of the older people, is frequently associated with a reduction in the quality of life and mobility. Falls often are the basis of reduced mobility and ability to perform the common functions of daily life and the increase in the number of institutionalizations. Moreover, the reduction of muscle mass, associated with altered muscle composition, fat and fibrous infiltration and alterations in innervations, and the increase in fat mass, have a synergistic effect on the increase in cardiovascular risk. The aim of this review is to analyze the pathophysiological mechanisms underlying the frailty syndrome and its association with sarcopenia and osteoporosis, and investigate possible intervention measures.

## Introduction

Musculoskeletal aging is a major public health interest and is strain typical of the demographic changes in the population. It is associated with high risk of falls, loss of autonomy in elderly people and institutionalizations with small health outcomes. This condition is therefore correlated with high morbidity and health care rates (1, 2).

Indeed, world population is aging and, worldwide, individuals over 60 are estimated to increase from 841 million in 2013 to more than 2 billion by 2050, with a proportional gain from 11 to 22%. However, often the increase in life expectancy is not an increase in “healthy life” expectancy, and these additional years are loaded with scarce health and disability (3).

Musculoskeletal aging has many causes, including age-related changes in body composition, inflammation, and hormonal imbalance. Furthermore, sarcopenia and osteoporosis are linked and commonly associated with aging, often leading to a frailty syndrome.

Frailty is a physical condition, typically observed in elderly people, characterized by a gradual and growing loss in the function or reserves of multiple physiologic systems, which increased vulnerability and inability to maintain or recover homeostasis after a destabilizing occurrence, such as fever, infection, surgery, falls, and homeostasis changes due to pharmacological therapies (4, 5). Then, frailty can be considered a biologic condition characterized by low resistance and response to stressors, as a consequence of a general decline which includes multiple systems and organs. The clinical signs of frailty are: body weight loss, sarcopenia, osteoporosis, declined physical activity, reduced balance and gait speed, reduced cognitive function, and alterated state of nutrition. Thus, frailty determines a high risk for reduced activities of daily living, for cardiovascular diseases, cancers, falls, limited mobility, and increases risk of hospitalization and mortality (6).

Fried et al. proposed a “physical” phenotype of frailty, consisting of five features for identification of frailty syndrome: weakness (evaluated by grip strength), slowness (evaluated by gait speed), reduced attitude to the physical activity, reduced energy (self-reported), and involuntary body weight loss. The presence of one to two features points a pre-frail condition while three or more indicates a frailty syndrome (7). Since frailty, defined by these criteria, has been correlated with adverse health outcomes (7), other more simplified models of frail phenotype were later developed using data from the Study of Osteoporotic Fractures and the Three-City Study. Their predictive potencies for disability and mortality were either similar or improved compared to the original Fried model of frailty (8, 9).

On the other hand, a “multi-domain” of frailty phenotype exists, proposed by Rockwood et al. (10) who developed the Frailty Index (FI), calculated from an extensive questionnaire of diseases and ill-health. FI is based on identified deficits in several domains such as cognition, mood, motivation, communication, mobility, balance, activities of daily living, nutrition, social resources, and several other comorbidities. FI is reported as a ratio of prevalent deficits to the total number of potential deficits, and the greater the rate of deficit a subject has, the more likely this subject is to be frail. This index is considered to be highly predictive of high risk of mortality and institutionalization (10).

A reduction of muscle mass and strength with a corresponding increase of fat mass in the elderly might synergistically increase the risk of cardiovascular diseases (11). In fact, during aging, fat mass increases and its distribution changes with a decrease in subcutaneous fat and an increase of visceral fat, leading to a new nosographic entity named sarcopenic obesity (3).

**Figure 1:**
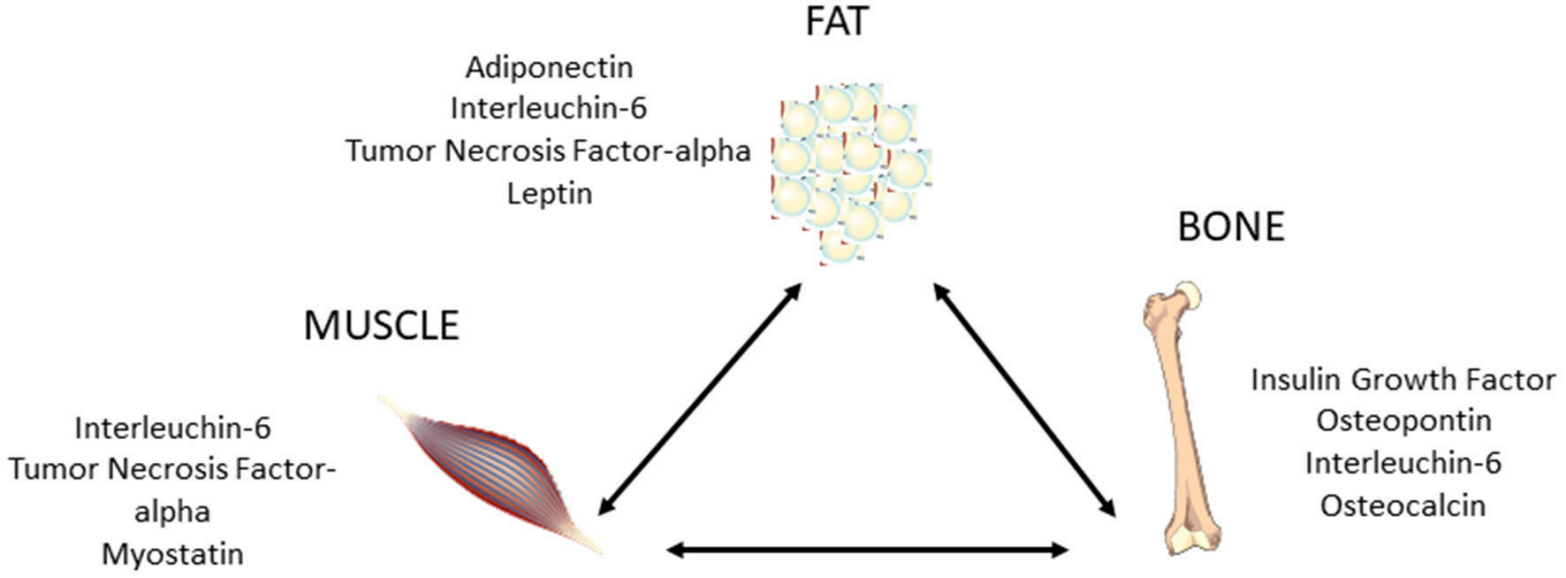
Physiopathological basis of frailty. Aging, reduced physical activity, nutrients intake, hormone imbalance, and chronic inflammation worsen pathological conditions as sarcopenia, obesity, and osteoporosis, which in turn lead to frailty syndrome.

The alterated fat distribution observed in aged people and/or in obese subjects is in fact characterized by intermuscular and intramuscular fat infiltration, both associated with a decline in muscle and mobility function and considered significant predictors of frailty and several comorbidities such as insulin resistance, diabetes, cardiovascular diseases, stroke, spinal cord injury, and chronic obstructive pulmonary disease (12). The mechanism(s) by which intermuscular and intramuscular fat negatively influences muscle function is actually unknown. However, the release of pro-inflammatory citokines from ectopic fat might provide this negative association (13), as well as the increased expression of Perilipin2 (Plin2), a protein associated with lipid droplet deposition, age and low muscle strength and thickness, both in humans and animal models (14) (Figure 1). In addition, as a consequence of fat muscle infiltration, motor units often undergo denervation and fast type II muscle fibers switch to slow type I fibers, leading to decreased muscle mass and strength (15, 16). A recent, interesting study shows that older patients who underwent extended high-intensity resistance training after hip fracture had improved quadriceps muscle mass and strength, while intramuscular fat remained unchanged (17).

Muscle mass and strength increase during late adolescence and early adulthood, and generally start to decrease from the fifth decade of life. In particular it decreases annually by 1–2% from the age of fifty, by 1.5% from the age of 50–60 and by 3% thereafter (3). Moreover, the decrease in muscle mass and strength negatively affect bone mass, which also declines during aging, causing osteopenia and osteoporosis. Aged, post-menopausal women have an increased risk of both osteoporosis and sarcopenia (1, 2), and present a loss of muscle performance more rapidly than men, suggesting a protective role of estrogens in the maintenance of muscle homeostasis beside their known role in skeletal health maintenance (18). In men, there is no androgen decline comparable to menopause, however, lower testosterone levels correlate with lower protein synthesis, loss of muscle mass, and sarcopenia (19).

Other important factors might affect muscle well-being are decreased protein intake and low-grade chronic inflammation, which often characterize aging. In elderly people, protein intake and protein synthesis are reduced and the production of pro-inflammatory factors is increased (18), and this low-grade inflammation further contributes to the anorexia of aging and correlates with reduced mobility and impaired cognitive function, representing an independent risk factor for disability (3).

Finally, a new interdisciplinary field named “geroscience” aims to understand the relationship between aging and chronic age-related diseases and geriatric syndromes. It is based on epidemiological evidence and experimental data that aging is the major risk factor for such pathologies, and assumes that aging, age-related diseases and geriatric syndromes share a common set of basic biological mechanisms. Geroscience assumes that an individual will follow an accelerated or de-accelerated aging process through his/her genetic background, interacting lifelong with environmental and lifestyle factors. It is clearly urgent to identify markers capable of distinguishing between biological and chronological age to identify subjects at higher risk of developing unhealthy aging. Recently, some authors have proposed the use of DNA methylation, N-glycans profiling and gut microbiota composition over the available disease-specific markers (20).

The aim of this review was to analyze reciprocal pathophysiologic relationships between the frailty syndrome, sarcopenia and osteoporosis. We performed Medline searches on the pathophysiology of the aforementioned geriatric syndromes, we describe common/joint disease mechanisms and present the concept of sarcopenic obesity. The final section of our review is dedicated to potential intervention measures.

## Sarcopenia and Osteoporosis as Concequences of an Altered Muscle-Bone Cross-Talk

Interestingly, data from many studies show that frailty is strictly associated with sarcopenia, osteopenia or osteoporosis, and falls (6), and these studies show that balanced physical activity and diet interventions are the focus of treatment, even though the treatment strategy depends on the specific frailty domain shown by the subject (3).

Several studies also show the correlation between low BMD and sarcopenia in both men and women (21). In the European Male Aging Study, in which 679 men aged 40–79 years were evaluated, sarcopenia was associated with osteopenia and osteoporosis (22), and similarly, high lean muscle mass and strength were positively associated with BMD. Whereas, sarcopenia was associated with low BMD and osteoporosis in a study of 17,891 subjects from various ethnicities (23). Moreover, a recent, interesting study by Locquet et al. showed, in a population of 232 elder people (age>75 years) of both sexes, that the decline in muscle performance was related to the decline in bone microarchitecture, and that subjects with incident sarcopenia had an approximately 5-fold increased risk of concomitantly developing osteoporosis, showing a dynamic relationship between impaired muscle and bone health, with an obvious association between the concomitant incidences of osteoporosis and sarcopenia (24). Finally, several studies showed that sarcopenia is an independent predictive factor of high fracture risk besides BMD and other clinical conditions (25), and that an association exists among sarcopenia, risk of falls and osteoporotic fractures (26–28). It is clear that the two conditions, sarcopenia and osteoporosis, are closely correlated, and that their combination leads to exacerbation of negative health effects and to frailty syndrome development (29).

Skeletal muscle is the body's scaffolding and allows movements and locomotion. Skeletal muscle can be affected by aging, low nutrition, disuse, inflammation, and hormone imbalance, that lead to loss of muscle mass and strength, a condition named “sarcopenia,” which is associated with frailty, cachexia, osteoporosis, metabolic alterations, and mortality. Like frailty, sarcopenia is strongly associated with loss of function and negatively influences people's ability for independent living, that might determine isolation and cognitive alterations, with an increase in the assistance care costs (3).

In 1989, Rosenberg first proposed the term “sarcopenia” (from the Greek “sarx” for flesh and “penia” for loss) to define the typical age-associated decrease in muscle mass (30), but during the last few decades its definition has been enlarged to include reduced muscle mass and reduced muscle function, and the consensus definition of sarcopenia is still under debate. Low lean mass, muscle strength and weakness are the main criteria considered to define sarcopenia proposed by the European Working Group on Sarcopenia in Older People (EWGSOP) and The Foundation for the National Institutes of Health (FNIH) Sarcopenia Project. Other proposed criteria include those from International Working Group (IWG), European Society for Clinical Nutrition and Metabolism Special Interest Group on cachexia-anorexia in chronic wasting diseases (ESPEN) and Society of Sarcopenia, Cachexia, and Wasting Disorders (SCWD) (31). Regardless of the definition, all scientific societies agree that the preservation of muscle strength and power with advancing age is of high clinical significance. However, despite the preponderance of scientific investigations that have continued to focus primarily on determinates of skeletal muscle size, recent longitudinal and intervention-based studies have clearly demonstrated that muscle atrophy is a relatively small contributor to the loss of muscle strength, and that exogenous supplementation of androgens or growth factors have yielded an increase in muscle mass but only marginally improved muscle performance. Then, on the basis of these observations, in 2008 Clark and Manini proposed the term “dynapenia” (*dyna* refers to “power, strength, or force” and *penia* refers to “poverty”) to define the age-related loss of muscle strength and power (32). Of course, dynapenia both in association with sarcopenia and as an independent factor, increases the risk of poor physical performance, disability and even death.

The pathophysiological basis of muscle mass and strength loss include a variety of factors and pathways, such as environmental factors, hormonal alterations, motor-neurons and muscle fibers loss, decreased protein synthesis and/or increased protein catabolism, activation of inflammatory pathways, reduction in satellite cell counts, and mitochondrial dysfunction and/or reduction (31).

**Figure 2:**
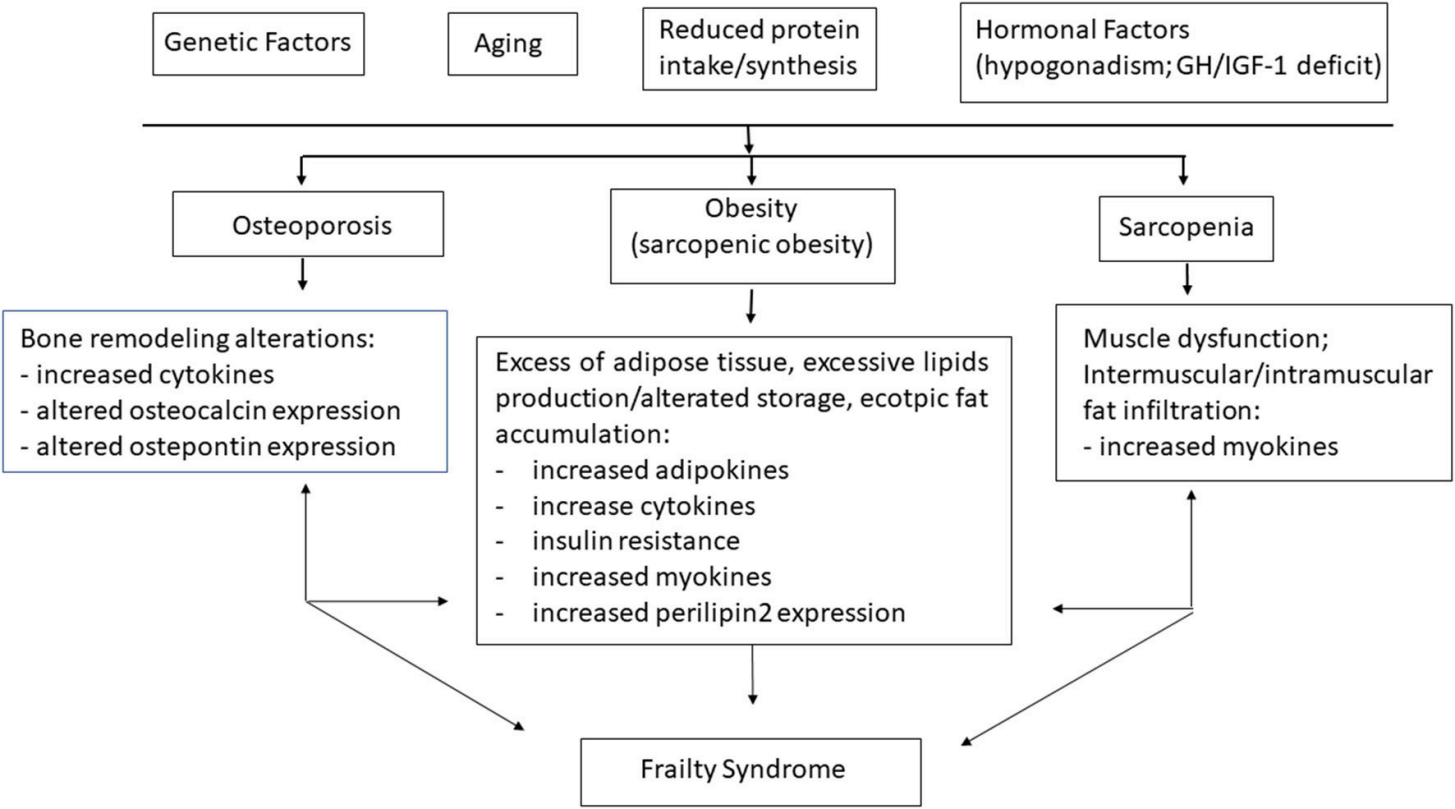
Schematic representation of molecular basis of the complex fat-bone-muscle cross-talk.

**Figure 3:**
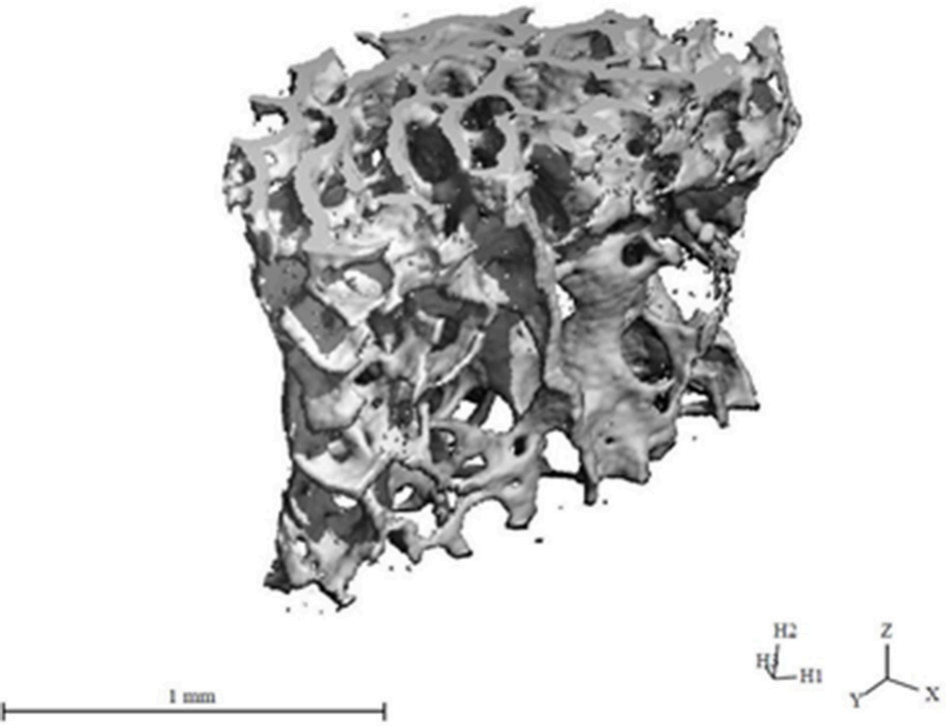
MicroCT image of the trabecular structure of a vertebral body of a C57Bl/6J mouse aged 12 month.

During aging, muscle fibers decrease in size and number and alterations in skeletal muscle composition occur. As mentioned above, an increased fat infiltration in skeletal muscle is described, which significantly alters muscle quality and performance, and leads to a sarcopenic obesity (33–35). The prevalence of obesity in association with sarcopenia is increasing in adults over the age of 65 and older, leading to a high risk of synergistic complications from both sarcopenia and obesity (36). In sarcopenic obesity the excess of adipose tissue determines a dysregulated production of several adipokines which, in association with senescent cell- and immune cell-derived cytokines, create a local pro-inflammatory status. In addition, obese adipose tissue, through the excessive lipids production and their alterated storage, favors fat muscle infiltration and insulin resistance leading to pro-inflammatory myokines secretion, which in turn induces muscle dysfunction by auto/paracrine manner. Finally, these myokines, by endocrine manner, exacerbate adipose tissue inflammation and support chronic low-grade systemic inflammation, establishing a detrimental vicious circle triggering and maintaining sarcopenic obesity development (37). The increased body and muscle fat are associated with insulin resistance and low-grade chronic inflammation, with an increase in many specific and unspecific inflammatory parameters, like C reactive protein (CRP), fibrinogen, interleukin-6 (IL-6), and tumor-necrosis-factor alpha (TNF-α), which lead to the decrease in both muscle mass and strength, and to bone loss (35) (Figure 2). Moreover, an alteration in mesenchymal stem cell differentiation is observed, that is characterized by a high differentiation in adipocytes, which leads to a reduced muscular renewal (35) and Figure 3.

Also, sex steroids are involved in the pathogenesis of sarcopenia. In fact, the reduced estrogen levels after menopause amplify the increase in inflammatory markers (IL-6 and TNF-α), and since myocytes expressed estrogen-β receptors, a direct effect of estrogens on muscle mass has been proposed (38, 39). However, conflicting data exist about hormone replacement therapy (HRT) and its possible use for the prevention of the musculoskeletal aging (40). Finally, a decline in androgen levels may also play a role in the pathogenesis of sarcopenia, both in men and women (41).

Mitochondrial reactive oxygen species (mtROS) are tightly linked to oxidative stress in age-related muscle mass and strength. The deposition of mtROS in aged muscle determines tissue damage, muscle atrophy, muscle dysfunction, and increases in fibrous tissue (42). Moreover, mitochondria act directly on apoptosis, and their alterations and mtROS promotes cell degradation, the reduction of muscle fibers, and muscle atrophy (43). Also, an increased mitophagy has been associated with muscle atrophy (44).

Finally, myostatin, which is a well characterized myokine and a member of the transforming growth factor-β superfamily, negatively modulates muscle mass and growth and, interestingly, increased myostatin levels appear to be associated with aging (45). Indeed, several studies reported that myostatin levels were increased in frail elder women and were inversely correlated with muscle mass (46–48). However, further studies are needed to better understand the relationship between myostatin and aging.

During aging, bone remodeling is reduced, leading to a negative bone balance and increasing the incidence of age-associated bone alterations, such as osteopenia and osteoporosis. In particular, osteopenia is a clinical term used to describe a decrease in bone mineral density (BMD) below normal reference values, yet not low enough to meet the diagnostic criteria to be considered osteoporotic while osteoporosis is a skeletal metabolism alteration that causes a loss of bone mineral density and quality. This leads to bone fragility and high fracture risk, and osteoporotic fractures are associated with high morbidity and mortality (21).

The incidence of osteoporotic fractures increases with age, and actually, in women over 80 the incidence of hip fractures is 30%, while the incidence of vertebral fractures is more than 40% (21). Then, elderly patients with osteoporotic fractures should be considered as frail subjects, with low post-fracture outcomes that lead to functional decline, loss of quality of life, and increased mortality, for the next 10 years after the fracture (49). Moreover, osteoporosis is often associated with sarcopenia, with similar consequences, such as physical impairment, institutionalization, and depression, all conditions that increase morbidity and mortality (7).

During the last decade, bone and muscle were increasingly recognized as interacting tissues, not only because of their local proximity and their integrated function for locomotion, but were recognized also as endocrine target organs and endocrine organs themselves (50–52). In fact, the two tissues interact by paracrine and endocrine signals and modulate their development and function from intrauterine life to old age, and a linear relationship between BMD and muscle mass at various ages exists (52–54).

During growth, bone mineral content and femoral circumference closely correlate with muscle tissue, and several evidences suggest that osteoporosis and sarcopenia present common pathophysiological factors, including hormonal imbalance, increased inflammatory cytokine activity, release of tissue-specific molecules, nutritional changes, and physical impairment (53–56). The muscle–bone cross-talk is supported by preclinical and clinical data (Figure 3), showing the presence of many tissue-specific factors released by muscle that modulate bone, such as insulin-like growth factor-1 (IGF-1), fibroblast-growth factor-2, IL-6, IL-15, myostatin, osteoglycin, irisin, and osteoactivin (52). Interestingly, many factors such as myostatin, TNF-α, IL-6, and ROS that, as described above, are involved in the pathogenesis of sarcopenia are also regulators of bone remodeling, and thus are relevant for osteoporosis (56). However, actually there exists limited data about the modulation of bone on muscle, and both osteoblasts and osteocytes were shown to produce specific molecules, including prostaglandin E_2_, osteocalcin, and IGF-1, which might impact skeletal muscle cells (52).

Finally, adipose tissue also interacts with bone and muscle, and obesity, sarcopenia, and osteoporosis could concomitantly exist. The increase in total and/or abdominal fat observed in obese subjects determines low chronic inflammation and hormonal imbalance which negatively affect both muscle and bone (50, 57). Indeed, people affected by sarcopenic obesity have a high risk of osteoporosis and fragility fracture, as well as other metabolic alterations resulting from changes in their body composition closely associated with high morbidity and mortality. These considerations of course emphasize the importance to strictly monitor bone health in sarcopenic obese subjects, mostly during aging (57).

## Do Sarcopenia and Osteoporosis Lead to Frailty Syndrome Development?

Frailty is often discovered by maladaptive response to stressors, causing functional decline and other serious adverse health outcomes (4). During the last decade, a large amount of data suggest many causes for the pathogenesis of the frailty syndrome, such as chronic inflammation, musculoskeletal and endocrine system alterations, nutritional changes, and physical impairment, leading to a vicious cycle characterized by a progressive muscle and bone loss, as well as fat gain (28) (Figure 1).

In particular, chronic inflammation is a key factor that contributes to frailty, both directly and indirectly through other intermediate mechanisms (58). The relationship between frailty and inflammatory markers is well known, and many studies conducted in elderly people support the effect of chronic inflammation and immune activation on the frailty syndrome development (59–61). Inflammatory molecules directly contribute to frailty or indirectly through its detrimental effects on musculoskeletal metabolism and endocrine system (62).

Sarcopenia and osteoporosis are major contributors to disability and frailty. The age-related chronic inflammation, often indicated as “inflammaging,” leads to the decrease in both muscle mass and strength and bone loss, such as sex steroids and GH decline (35). In 2000, Franceschi et al. described the phenomenon of inflammaging as part of the spectrum of immunosenescence, leading to muscle and potentially bone loss (63). Inflammaging is believed to be a consequence of a cumulative lifetime exposure to antigenic load caused by both clinical and sub-clinical infections, as well as from exposure to non-infective antigens. The consequence is an inflammatory response, tissue damage and the production of ROS which result in the release of additional cytokines, determining a vicious cycle driving immune system remodeling and favoring a chronic pro-inflammatory state (63).

Sarcopenia and osteoporosis are also promoted by an increase in body fat and alteration in its distribution. With age, subcutaneous fat decreases despite the increase of visceral fat and fat infiltration of muscle fibers (3). The age-related increased body fat and muscle fat infiltration promote insulin resistance and inflammation that, through a vicious loop mechanism, determines muscle and skeletal metabolism alterations and dysregulation in mesenchymal stem cell differentiation leading to sarcopenia and osteoporosis (35). Obesity is also associated with sarcopenia, osteoporosis and frailty in both men and women as demonstrated by several studies, likely due to adipose tissue involvement in the complex bone–muscle interaction (50). In this view, obesity, sarcopenia, and osteoporosis could concomitantly exist, and the increase in total and/or abdominal fat and the excess fatty acids in the muscle fibers have also been shown to interfere with normal cellular signaling and favor inflammation and hormonal imbalance affecting both muscle and bone (50, 57). Further, sarcopenic obese subjects have a high risk of osteoporosis, fragility fractures and chronic metabolic disorders, resulting from the changes in their body composition (58).

Finally, sex steroids and IGF-1 are essential for bone and muscle metabolic regulation (62) and, indeed, the role of sex hormones in the pathogenesis of sarcopenia and osteoporosis has been well documented. The rapid drop in estrogens after menopause and the gradual decrease of androgens in older men result in decreased bone and muscle mass, with an increased risk of fragility fractures and sarcopenia in both genders but with a different timing; at the same time the low sex steroid levels determine an increase of the inflammatory markers which are linked to both sarcopenia and osteoporosis (38, 39). Circulating levels of dehydroepiandrosterone sulfate (DHEA-S) and IGF-1 are also significantly lower in frail older adults as compared to non-frail individuals, and many other hormones, such as cortisol and vitamin D, have also been associated with sarcopenia and frailty in the elderly, suggesting a potential impairment of the GH–IGF-1 somatotropic axis, the hypothalamic–pituitary–adrenal axis, and other hormones on the basis of frailty syndrome (62).

The altered cellular and molecular signals and functions described above, which lead to sarcopenia, osteoporosis, and inflammation, seem to be linked in a circle manner and probably represent the common denominator favoring frailty and unhealthy aging. However, further clinical and biological investigations are needed to better understand the complex multifactorial etiology of frailty.

## Intervention Measures

Nutritional intervention and physical activity are two pivotal measures for the prevention and treatment of sarcopenia, and they act in a synergistic manner.

Physical exercise in middle age seems to reduce the development of sarcopenia in older adults and it is also the primary measure for maintaining muscle mass and strength, and performance in elderly, as has been shown in the ROAD study, an observational study conducted on 1773 older adults, followed for 4 years (64). The aim of this study was to investigate the possible association of physical activity of daily living with the incidence of certified need of care in the national long-term care insurance system in elderly Japanese population-based cohorts, showing that physical dysfunction in daily living is a predictor of the occurrence of certified need of care (64). Moreover, a recent meta-analysis, including clinical trials on varied physical activity interventions for sarcopenia, showed a statistically significant association between physical activity and sarcopenia and documented its protective role against sarcopenia development, as well as heart diseases, diabetes, osteoporosis and pulmonary diseases (65). In fact, physical activity improves body composition by increasing muscle mass, reducing body fat, and improving muscle strength and endurance. In addition, physical activity can also modulate immune function and the cardiovascular system and, thus, it should be considered an essential measure of therapeutic strategies of age-related sarcopenia. Aerobic exercise improves mitochondria functions, aerobic capacity, metabolic regulation, and cardiovascular function. Also, aerobic exercise decreases the expression of catabolic genes and increases muscle protein synthesis (66–68). Resistance exercise prevents muscle wasting by stimulating muscle hypertrophy and increases muscle strength by regulating the protein metabolism balance (69). However, no single type of exercise but the combination of aerobic and resistance exercises should be preferred to prevent and treat the potential molecular mechanisms of age-related sarcopenia (70).

During aging, energy requirements decline, as do food and energy intake (71). Reduced food intake in elderly determines weight loss, with consequential decrease of muscle mass and strength and physical impairment (72). The importance of balanced nutrition in older adults has been recognized for a long time, but only recently have studies been designed to explore the effects of nutrition on muscle mass and physical performance (73). These studies suggest that diet has an important role in the prevention and management of sarcopenia and several kinds of interventions have been tested. The nutrients that have been most closely linked to the development of sarcopenia and frailty are protein, vitamin D, antioxidant nutrients (like carotenoids, selenium, and vitamins E and C), and long-chain polyunsaturated fatty acids (74). The few studies performed to date seem to indicate that there is a protective role of protein supplementation against frailty syndrome and it is tempting to suggest daily 30 g protein supplements help to prevent frailty. However, it is well established that excess protein can also be harmful; therefore, specific individual characteristics should be considered before prescribing these supplements. On the other hand, the relevance of other nutritional interventions, such as vitamin D, omega-3, and medium-chain triglycerides, is much more scarcely researched in the literature (75). Therefore, new clinical trials are necessary to carry out effective nutritional interventions to prevent frailty development.

Pharmacological therapies for the prevention and treatment of frailty are represented by drugs used to control both osteoporosis and sarcopenia. Anti-osteoporotic agents are used to increase bone mass and reduce fracture risk, such as bisphosphonates, denosumab, and teriparatide, associated with calcium and vitamin D supplementation. Advanced age is associated with increased signaling through extrinsic and intrinsic apoptotic pathways in skeletal myocytes, favoring the development of sarcopenia. Several preclinical studies suggest that myonuclear apoptosis might provide a selective biological target for the development of preventive and therapeutic interventions against sarcopenia. Many strategies, such as calorie restriction, exercise training, and drugs determine the down-regulation of myocyte apoptosis counteract the development or worsening of sarcopenia and muscle dysfunction (76). In particular, to prevent and treat sarcopenia, the appropriate pharmacological strategy might include myostatin inhibitors and type II activin receptor inhibitors; follistatin; testosterone or selective androgen receptor modulators (SARMs); angiotensin-converting-enzyme (ACE), inhibitors, and ghrelin mimetics (77). Other hormonal therapies, such as GH, IGF1, and estrogens, have also been experienced, but no evident beneficial effects have yet been demonstrated (78). The approach to increase muscle mass is the same for either young athletes or elderly individuals, however, in elder adults the need for a prolonged treatment makes it difficult to treat sarcopenia because of compliance and safety. Finally, since preclinical and clinical studies have demonstrated an increased number of senescent cells in the bone microenvironment during aging, with a consequential alteration in bone remodeling due to an increased secretion of inflammatory markers, a potential approach might either eliminate senescent cells or impair the production of their inflammatory factors, representing a novel therapeutic strategy to prevent multiple age-related diseases (79).

To treat and/or prevent frailty damages, early identification of people at risk of sarcopenia and osteoporosis is important. The European Working Group on Sarcopenia in Older People 2 (EWGSOP2) and the International Conference on Frailty and Sarcopenia Research (ICFSR) task force have recently produced a consensus and evidence-based clinical practice guidelines for its definition, screening, diagnosis and management (80, 81). Moreover, the ICSFR task force evaluated the evidence behind several topics (definition of sarcopenia, screening and diagnosis of sarcopenia, physical activity prescription, protein supplementation, vitamin D supplementation, anabolic hormone prescription, medication under development), considering the quality of evidence, the benefit-harm balance of treatment, patient preferences/values, and cost-effectiveness, and strongly recommend treatment of sarcopenia with prescribed resistance-based physical activity and conditionally recommended protein supplementation or a protein-rich diet (81).

## Conclusions

World population is aging and the increase in life expectancy is often unhealthy. In particular, musculoskeletal aging, which leads to sarcopenia and osteoporosis, has several causes such as changes in body composition, inflammation, and hormonal imbalance. Sarcopenia, osteoporosis, and more frequently, sarcopenic obesity are commonly associated with aging and frequently closely linked each other, often leading to the development of a frailty syndrome. Frailty syndrome favors an increased risk of loss function in daily activities, for cardiovascular diseases, cancers, falls, and mortality. As the number of elderly people continues to increase, it is important to identify people at risk of frailty early and to treat and/or prevent its damages, developing interventions that can promote a “successful aging.” The complexity and heterogeneity of frailty syndrome requires a multidimensional clinical approach based on healthy nutrition, psychosocial well-being, regular physical exercise, and pharmacological measures, which seem to prevent and control chronic diseases affecting both life expectancy and quality of life, thereby reducing mortality. Of course, new basic and clinical studies are necessary to better understand the complex pathophysiological mechanisms leading to frailty and to carry out effective measures of interventions to prevent its development and treat its damages.

## Author Contributions

EAG, PP, and SM equally contributed to the preparation of the manuscript.

### Conflict of Interest Statement

The authors declare that the research was conducted in the absence of any commercial or financial relationships that could be construed as a potential conflict of interest.
